# Palo: spatially aware color palette optimization for single-cell and spatial data

**DOI:** 10.1093/bioinformatics/btac368

**Published:** 2022-06-01

**Authors:** Wenpin Hou, Zhicheng Ji

**Affiliations:** Department of Biostatistics, Johns Hopkins University Bloomberg School of Public Health, Baltimore, MD 21205, USA; Department of Biostatistics and Bioinformatics, Duke University School of Medicine, Durham, NC 27710, USA

## Abstract

**Summary:**

In the exploratory data analysis of single-cell or spatial genomic data, single-cells or spatial spots are often visualized using a two-dimensional plot where cell clusters or spot clusters are marked with different colors. With tens of clusters, current visualization methods often assign visually similar colors to spatially neighboring clusters, making it hard to identify the distinction between clusters. To address this issue, we developed Palo that optimizes the color palette assignment for single-cell and spatial data in a spatially aware manner. Palo identifies pairs of clusters that are spatially neighboring to each other and assigns visually distinct colors to those neighboring pairs. We demonstrate that Palo leads to improved visualization in real single-cell and spatial genomic datasets.

**Availability and implementation:**

Palo R package is freely available at Github (https://github.com/Winnie09/Palo) and Zenodo (https://doi.org/10.5281/zenodo.6562505).

**Supplementary information:**

Supplementary data are available at *Bioinformatics* online.

## 1 Introduction

Data visualization is a key step in exploring the underlying structure of single-cell and spatial genomic data. For single-cell sequencing data [e.g. single-cell RNA-seq (Tang *et al.*, 2009)], cells are commonly projected into a low-dimensional space using methods such as Uniform Manifold Approximation and Projection (UMAP, Becht *et al.*, 2019) or t-Distributed Stochastic Neighbor Embedding (t-SNE, Van der Maaten and Hinton, 2008) and visualized by a 2-D scatterplot where the two axes represent two reduced dimensions. Cells with the same cell type or cluster are shown with the same color. For spatial transcriptomics data (Ståhl *et al.*, 2016), spatial spots are visualized by a 2-D spatial map where the two axes represent the two spatial coordinates of the tissue slide. Similarly, spots with the same cluster are shown with the same color. The visualization guides downstream analyses such as cell type identification (Abdelaal *et al.*, 2019) and trajectory reconstruction (Hou *et al.*, 2021; Ji and Ji, 2016; Trapnell *et al.*, 2014).

**Fig. 1.**
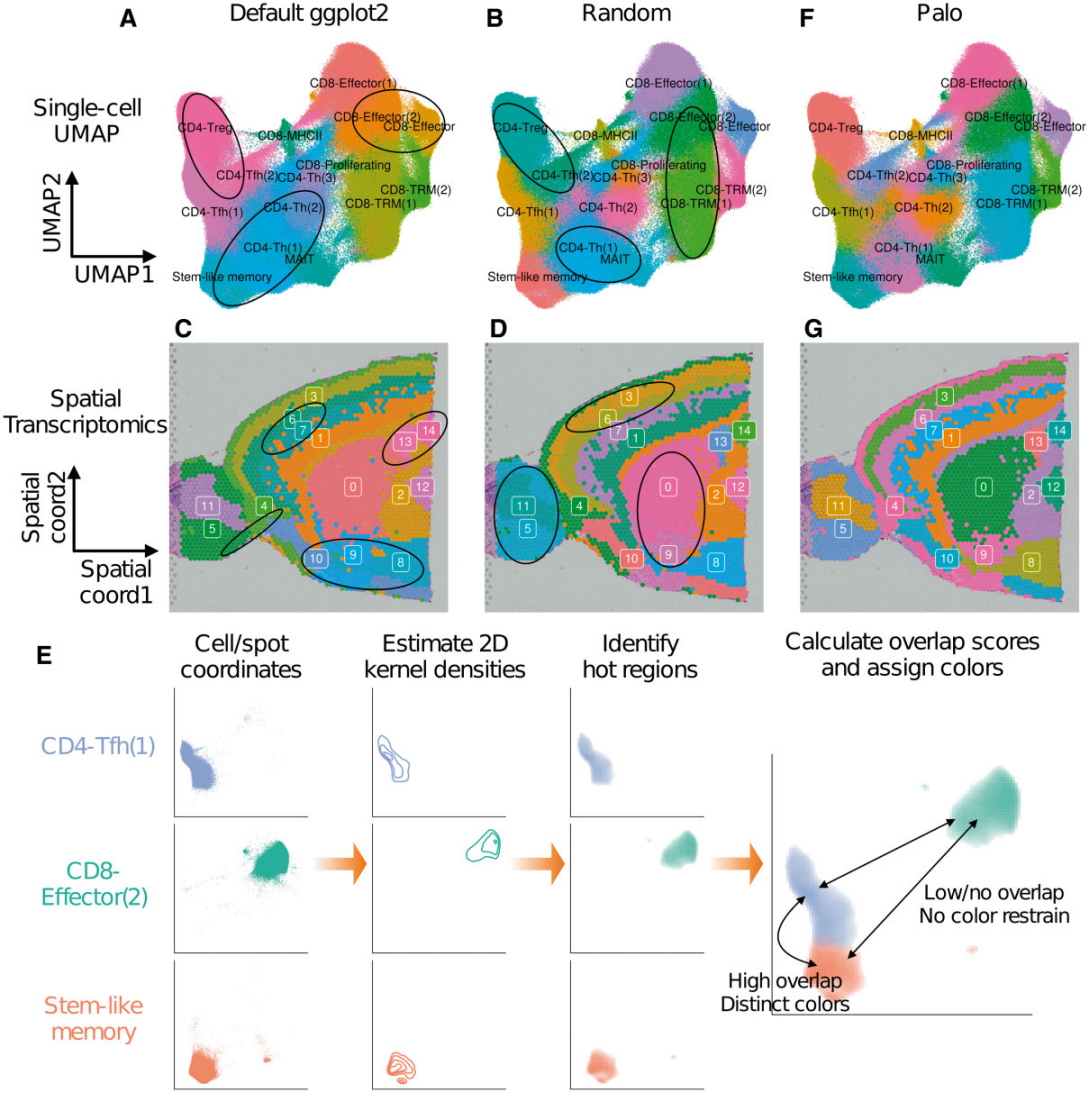
Visualization of single-cell RNA-seq data with default ggplot2 palette (**A**) or a randomly permuted palette (**B**). Neighboring clusters with visually similar colors are circled. Visualization of spatial transcriptomics data with default ggplot2 palette (**C**) or a randomly permuted palette (**D**). Neighboring clusters with visually similar colors are circled. (**E**) Schematic of Palo. (**F**) Visualization of single-cell RNA-seq data with Palo palette. (**G**) Visualization of spatial transcriptomics data with Palo palette

In many cases, cells or spots are grouped into tens of clusters to reflect their heterogeneity, thus tens of different colors are needed to visualize the different clusters. This will inevitably lead to similar colors in the color palette that are hard for human eyes to perceive and differentiate. As existing methods [e.g. ggplot2 (Wickham, 2016)] assign colors to clusters either alphabetically or in a random order, it is highly likely that some spatially neighboring clusters are assigned similar colors that are hard for human eyes to differentiate. Figure 1A shows an example of visualizing a single-cell RNA-seq dataset with different T cells subsets (Caushi *et al.*, 2021). UMAP coordinates were obtained using the standard Seurat (Stuart *et al.*, 2019) pipeline and cell type information was from the original publication. The geom_point() function in ggplot2 R package (Wickham, 2016) was used to generate the plot with the default color palette and settings. Multiple neighboring clusters [e.g. CD4-Treg and CD4-Tfh(2)] share similar colors that are hard to differentiate. This problem cannot be solved by randomly permuting and reassigning colors to clusters (Fig. 1B). Figure 1C shows an example of visualizing Visium spatial transcriptomics data of a mouse brain (10X Genomics, 2020). Spot clusters were obtained using the standard Seurat (Stuart *et al.*, 2019) pipeline. The plot was generated using the SpatialDimPlot() function in Seurat R package (Stuart *et al.*, 2019) with the default color palette and settings. Similarly, there are neighboring clusters (e.g. clusters 8, 9, 10) that share similar colors and are not visually distinct. Randomly permuting and reassigning the color palette cannot resolve the issue (Fig. 1D).

This visualization issue may create false impressions of cell type abundances or spatial interactions between spot clusters. It cannot be directly addressed by existing visualization methods such as ASAP (Gardeux *et al.*, 2017), dittoSeq (Bunis *et al.*, 2021), SPRING (Weinreb *et al.*, 2018) and SCUBI (Hou and Ji, 2022) which focus on other aspects of visualization. A simple solution is to manually exchange the colors assigned to different cell clusters multiple times. However, this manual process is tedious and time-consuming when there are many colors to be exchanged or when each cell cluster is spatially close to numerous other clusters. Plus, the manual process cannot fit in automatic analysis pipelines or efficiently handle a large number of samples or datasets.

To address this issue, we developed Palo to optimize the color palette assignments to cell or spot clusters in a spatially aware manner. Palo first calculates the spatial overlap score between each pair of clusters. It then identifies a color palette that assigns visually distinct colors to cluster pairs with high spatial overlap scores (Fig. 1E). We applied Palo to both the single-cell RNA-seq dataset (Fig. 1F) and the spatial dataset (Fig. 1G). The results show that Palo resolves the visualization issue, and spatially neighboring clusters are assigned visually distinct colors. The optimized color palette by Palo improves the visualization and identification of boundaries between spatially neighboring clusters.

## 2 Materials and methods

The inputs to Palo are (i) the 2-D coordinates of cells or spots; (ii) a vector indicating clusters of the cells or spots; (iii) a vector of user-defined colors. For single-cell genomic data, the coordinates are usually obtained by dimension reduction. For spatial data, the coordinates are the spatial locations of spots in a tissue slide. The output of Palo is the optimized permutation of the user-defined input color vector assigned to the clusters.

The Palo method consists of the following steps. Step 1: for each cluster, a 2-D kernel density function [MASS::kde2d() in R] with 100 × 100 grid points is fitted using the 2-D coordinates of all cells or spots in the cluster. Step 2: for each cluster, all grid points with density values larger than a cutoff are treated as the hot grid points. To identify the cutoff, the cluster labels for all cells or spots are randomly permuted once, and the 2-D kernel density function is refitted for each permuted cluster. For each cluster, the cutoff is the 95 percentile of the density values across all grid points obtained in the permutation. Step 3: for a pair of clusters *a* and *b*, an overlap score is calculated as the Jaccard index $J_{a,b}=|S_{a}\cap S_{b}|/|S_{a}\cup S_{b}|$, where *S_a_* and *S_b_* are the sets of hot grid points of *a* and *b*, respectively. Step 4: for a pair of colors *e* and *f*, the color dissimilarity $D_{e,f}$ is defined as the Euclidean distance between the red, green, and blue (RGB) values of the two colors. Different weights can be specified for each of RGB to better match how human eyes perceive the actual colors. For colorblind-friendly visualizations, Palo can also convert the colors to simulate how the colors are perceived by people with color-blindness, and the RGB distances will be calculated with the converted colors. Step 5: let *P* be a permutation of the user-defined color vector and *P_k_* be the color assigned to cluster *k*. A color score is defined as $\sum_{a\in C,b\in C}J_{a,b}\times D_{P_{a},P_{b}}$, where C=1,2,…,C and *C* is the total number of clusters. Palo finds *P* that maximizes the color score. To do that, Palo first randomly permutes the user-defined color vector multiple times (1000 times by default) and finds the initial permutation with the highest color score. It then fine-tunes the permutation by repeatedly exchanging colors between a pair of randomly selected clusters. If the exchange results in an increased color score, the exchange is kept. The exchange is repeated multiple times (2000 times by default). An early stopping rule is employed to stop the exchange process when the color score remains unchanged for several consecutive exchanges (500 consecutive exchanges by default). Supplementary Figure S1 shows how the color score changes with iterations for the two datasets analyzed in this study.

## 3 Implementations

Palo is implemented as an open-source R package. The package has one function, Palo(), that performs the color palette optimization. The following R command runs Palo:

pal <- Palo(position,cluster,palette)

Here, position is a cell by reduced dimension coordinate matrix with two columns (single-cell data) or a spot by spatial coordinate matrix with two columns (spatial transcriptomics data); cluster is a vector of cell or spot clusters; and palette is a user-defined color vector.

The output pal is a named vector of optimized color palette which can be directly fed into other functions in R for plotting. For ggplot2:

ggplot(…) + geom_point() +

scale_color_manual(values=pal)

For spatial maps in Seurat:

SpatialDimPlot(…) +

scale_fill_manual(values=pal)

## Funding

Z.J. was supported by the National Institutes of Health [1U54AG075936-01]. W.H. was supported by the National Institutes of Health [1K99HG011468]. W.H. would like to acknowledge Dr. Hongkai Ji, Dr. Stephanie C. Hicks and Dr. Andrew P. Feinberg for their mentorship.

*Conflict of Interest*: none declared.

## Data availability

The T cell single-cell RNA-seq dataset was obtained from Gene Expression Omnibus (GSE176022). The spatial transcriptomics dataset was obtained from 10X Genomics website (https://www.10xgenomics.com/resources/datasets/mouse-brain-serial-section-1-sagittal-anterior-1-standard-1-1-0).

## Supplementary Material

btac368_Supplementary_Figure_1
