# Supplementary figures and images for: Palo: spatially aware color palette optimization for single-cell and spatial data
Source: Bioinformatics. 2022 Jun 1;38(14):3654–6. doi: 10.1093/bioinformatics/btac368 (PMC9272793; doi:10.1093/bioinformatics/btac368)

**A**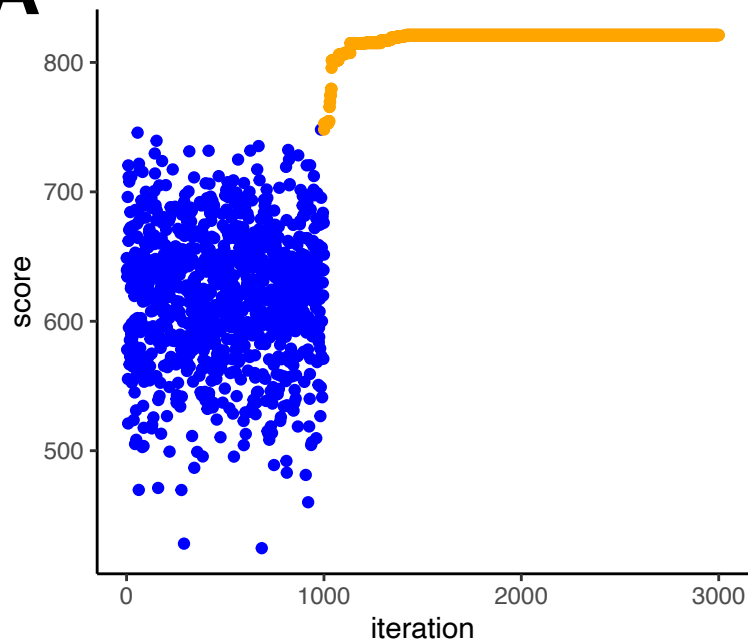

● initial permutation ● fine-tuning

**B**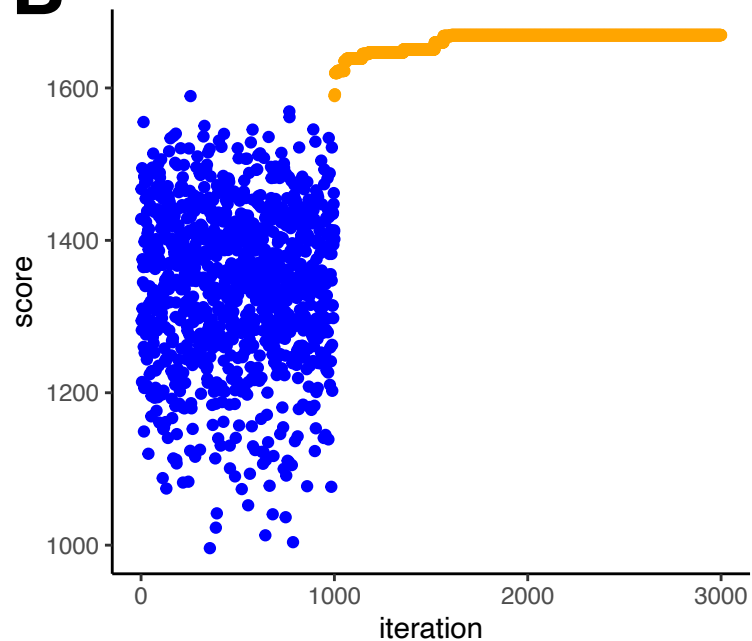

● initial permutation ● fine-tuning

Supplement: btac368_Supplementary_Figure_1 [file btac368_supplementary_figure_1.pdf]
